# Quantification of ∆^9^-tetrahydrocannabinol, 11-OH-THC, THC-COOH, hexahydrocannabinol, and cannabidiol in human plasma and blood by liquid chromatography–tandem mass spectrometry

**DOI:** 10.1093/jat/bkae094

**Published:** 2024-12-05

**Authors:** Marion Pavlic, Carolin Innerhofer, Florian Pitterl

**Affiliations:** Institute of Legal Medicine, Medical University of Innsbruck, Muellerstrasse 44, Innsbruck 6020, Austria; Institute of Legal Medicine, Medical University of Innsbruck, Muellerstrasse 44, Innsbruck 6020, Austria; Institute of Legal Medicine, Medical University of Innsbruck, Muellerstrasse 44, Innsbruck 6020, Austria

## Abstract

Ongoing legalization of cannabis for recreational use contributes to increasing numbers not only of incidents of driving under the influence, but within all forensic fields. In addition, newly emerging cannabinoids such as hexahydrocannabinol (HHC) and the increasing use of cannabidiol (CBD) products have to be addressed. The aims of this study were first to extend laboratory analysis capacity for the “established” cannabinoid ∆^9^-tetrahydrocannabinol (THC) and its metabolites 11-OH-THC and THC-COOH in human plasma/blood, and second to develop analytical procedures concerning HHC and CBD. An LC–MS–MS method based on the available (low-end) instrumentation was used. Samples (250 µl) were prepared by protein precipitation and solid-phase extraction. Chromatographic separation was achieved on a reversed-phase C18 column within 15 min. Detection was performed on a 3200 QTRAP instrument (Sciex) in positive multiple reaction monitoring (MRM) mode. Matrix-matched six-point calibrations were generated applying deuterated internal standards for all analytes except HHC. The method was fully validated according to GTFCh guidelines. Linear ranges were 0.5–25 µg/l for THC, 11-OH-THC, HHC and CBD, and 2.0–100 µg/l for THC-COOH, respectively. Limits of detection and limits of quantification were 0.5 and 1.0 µg/l (THC, 11-OH-THC, HHC, CBD), and 2.0 and 4.0 µg/l (THC-COOH). Applicability of plasma calibrations to blood samples was demonstrated. Acceptance criteria for intra- and inter-day accuracy, precision, extraction efficiency, and matrix effects were met. No interfering signals were detected for 80 exogenous compounds. The presented method is sensitive, specific, easy to handle, and does not require high-end equipment. Since its implementation and accreditation according to ISO 17025, the method has proven to be fit for purpose not only in driving under the influence of drug cases but also within postmortem samples. Furthermore, the design of the method allows for an uncomplicated extension to further cannabinoids if required.

## Introduction

Psychoactive effects after consumption of “hemp” have been known for centuries, as well as its medicinal action [1]. The first isolated cannabinoids were cannabinol (CBN) and cannabidiol (CBD), presented in 1940; albeit both showed no obvious “marihuana activity” [2]. In the meantime, more than 60 cannabinoids have been isolated from plant species *Cannabis sativa* and thoroughly investigated [3]. In order to distinguish them from endocannabinoids, such as anandamide and 2-arachidonoyl glycerol [4], they are often called phytocannabinoids [5]. The primary—if not exclusive—target of endocannabinoids as well as psychoactive phytocannabinoids is the cannabinoid receptor type-1 (CB1). CB1 are G protein-coupled receptors that are densely expressed in the central nervous system [6, 7], but were also detected in peripheral tissues such as Leydig cells in testis [8] or in cardiac muscle [9, 10]. CB1 are believed to regulate cognitive processes as well as behavioral procedures such as locomotion, pain sensation, catalepsy, and regulation of body temperature [11]. CB1-specific binding patterns in brain were shown to be consistent with the known behavioral effects using cannabis products [12] and suggest that CB1 is involved in the CNS effects experienced by cannabis users [6].

It became common knowledge that delta9-tetrahydrocannabinol (THC, IUPAC classification (6aR,10aR)-6,6,9-trimethyl-3-pentyl-6a,7,8,10a-tetrahydro-benzo[c]chromen-1-ol) is the major psychoactive component in cannabis products [13], acting via CB1 receptors [4, 14, 15]. It may be mentioned that THC also targets CB2 receptors that show some homology to CB1 and are primarily located in peripheral tissue [16–18], and on immune cells such as B cells and macrophages [19]. It was shown that CB2 agonists lead to anti-inflammatory responses and improve symptoms of inflammatory diseases [20], and studies showed promising anxiolytic-like effects of CB2 agonists [21]. Both receptors are involved in pain regulation [22–24] and were studied for anticancer purposes [25].

After smoking marijuana, THC can be detected up to 5–8 h in plasma, depending on the frequency of cannabis use and the application route [26, 27]. However, heavy users may exhibit THC-levels in plasma for >24 h resembling concentrations that infrequent users show after acute cannabis use [28]. Metabolic pathways of THC in the human body have been extensively studied, revealing phase I and phase II metabolites such as the main psychoactive metabolite 11-hydroxy-delta9-tetrahydrocannabinol (11-OH-THC) [29–31], and the nonpsychoactive 11-nor-9-carboxy-delta9-tetrahydrocannabinol (THC-COOH) in urine with a urinary excretion half-life ranging from 0.8–9.8 days [32], as well as in plasma [26, 33]. After oral ingestion, very little unchanged THC was present in plasma [34], and 80–90% of THC metabolites are excreted within 5 days, as the bioavailability of THC is only 4–12%, as compared to 10–35% after smoking [35]. Explanations are acidic degradation in the gastrointestinal tract and extensive first-pass liver metabolism of THC, respectively [36].

Detection of THC and 11-OH-THC in plasma or blood proves impairment at the time of sample drawing, albeit the self-reported “high” effect after consumption seems to lag somewhat behind the measured plasma levels [37]. In contrast detection of the non-psychoactive THC-COOH only proves that consumption has taken place. This is true in general if substances and/or their metabolites are detected in urine, and not in blood/plasma samples.

Over the years, CBD (IUPAC classification 2-[(1 R,6 R)-3-methyl-6-prop-1-en-2-ylcyclohex-2-en-1-yl]-5-pentylbenzene-1,3-diol) also has gained more and more attention. CBD is a high potency CB1 antagonist [38] and mediates several other mechanisms, e.g. serotonin-receptor-related neurotransmission [39]. In 2020, the European Court of Justice stated that CBD extracted from plants should not be considered a drug under the 1961 United Nations Single Convention on Narcotic Drugs [40]. It has been studied for medical purposes, especially CNS disorders [41], and has gained importance as preparation Epidiolex/Epidyolex for treatment of seizures associated with several syndromes in children such as Lennox-Gastaut, Dravet, or tuberous sclerosis [42, 43]. Otherwise, it is widely promoted for having anti-inflammatory, relaxing, antidepressive, anxiolytic, and pain-relieving effects without any relevant side effects [44], albeit serious scientific studies showed that consumption of CBD is not risk-free, especially in combination with other applied drugs [45, 46]. Altogether, the sale of low-THC cannabis products, containing mainly CBD, has been steadily increasing in central Europe since a few years, starting in Switzerland and soon after in neighbouring countries as well, such as Italy and Austria [47], where currently increasing business volumes of about 1.4% per year are expected [48].

Recently also hexahydrocannabinol (HHC, IUPAC classification 6,6,9-trimethyl-3-pentyl-6a,7,8,9,10,10a-hexahydrobenzo[c]chromen-1-ol) has gained broader public attention. The term HHC commonly summarizes the two stereoisomers (9 R)-HHC and (9S)-HHC. It was first described as component in hemp extract as early as in 1940 [1], and further chemical characteristics of HHCs were specified soon after [2]. HHC and derivatives thereof were found to be potent CB1 agonists *in vitro* [49] as well as *in vivo* in rodents, displaying cannabimimetic effects in usual tests such as the tail-flick assay for antinociceptive responses, or hypothermic effects that are associated with CB1 receptor binding [50, 51]. This is not surprising, as HHC represents a hydrogenated analog of THC. However, it has been indicated that HHC derivatives exhibit a slower onset of action in comparison to THC, as shown in rodents after injection [50], albeit the CB1 binding affinity of THC was shown to be less potent than that of (9 R)-HHC with binding affinity constants of 27.3 nM and 19 nM, respectively [52].

After entering the drug market as alternative to THC in the USA, it was first identified in Europe in 2022 in a food product marketed as sleep aid [53]. In the meantime, it has been detected in hemp-derived resin [54], in vaping devices typically containing cannabis-oil cartridges [55] and in food products, so called edibles [53]. Semisynthetic large-scale production of HHC can be done using CBD as pre-precursor [56]. Usually, CBD undergoes acid-catalyzed intramolecular cyclization in a first step to delta8-THC as main product [57, 58]. In a second step, HHC is produced by catalytic hydrogenation of mainly delta8-THC, leading to different ratios of (9S)- and (9 R)-HHC depending on the catalyst used [54, 59], with (9 R)-HHC being the substantially more active isomer [60].

In the beginning in 2021, HHC was offered as legally available substance since it has not been clearly regulated by most national legislations, albeit it has been monitored as “new psychoactive substance” since 2022 within the EU [53]. However, HHC and its derivatives have been regulated since March 2023 by the Austrian New Psychoactive Substances Act [61] and by the Swiss Narcotic Act [62].

Cannabis consumption impairs driving skills [63, 64] and is associated with an increased risk of road traffic accidents [65]. However, in a more recent study, no significant impact on driving abilities was found in 33 volunteers after smoking of “CBD-rich” marijuana with a CBD concentration of 16.6% [66]. Consequently, it seems necessary to survey drivers participating in road traffic concerning possible drug consumption, especially cannabis products of all sorts. Furthermore, highly increasing numbers of driving under the influence of drugs (DUID) cases have been observed in recent years, showing an increase of ∼10% from 2019 to 2020 for cannabinoids [67], Austria being no exception with an estimated increase of 15% from 2017 to 2022 [68]. Main explanations are extended police and medical resources as well as improved training of police officers involved in vehicle and driver inspections. The number of DUID cases analyzed at the Institute of Legal Medicine in Innsbruck, covering Western Austria with an estimated population of 1.15 million, has multiplied by a factor of about 10 in the last 5 years. Not surprisingly, cannabis impairment was the predominant result among these cases, in accordance with previous studies [69, 70]. Furthermore, it can be expected that further legalization of cannabis consumption, e.g. as in Germany in 2024, will lead to yet another overall rise of cannabis positive cases. Accordingly, keeping pace with increasing sample numbers requires extended laboratory analytical capacity. Simultaneously, reasonably available resources have to be considered. This does not only apply exclusively for the “established” cannabinoid THC and its main metabolites 11-OH-THC and THC-COOH, but also for other emerging cannabinoids such as HHC and CBD, requiring adapted analytical procedures.

Our aim was therefore to establish an efficient and robust liquid chromatography–tandem mass spectrometry (LC–MS–MS) method based on easily available (low-end) instrumentation enabling the simultaneous determination of THC, 11-OH-THC, THC-COOH, HHC, and CBD in plasma and whole-blood. We are aware that a number of methodological papers for quantitative analysis of HHC and HHC-metabolites and/or other cannabinoids have been published by now. However, they are either covering a subset of analytes, e.g. focusing on HHC and its metabolites [71, 72], and/or apply GC–MS(–MS) techniques requiring derivatization steps prior to GC analysis [73, 74]. The presented method is fully validated, robust, efficient, and fit for purpose of unambiguous and reliable differentiation between traditional cannabis consumption and consumption of HHC and/or CBD products. Another aim of this study was to prove the applicability of the method not only in DUID cases, but also in postmortem samples, thus fully covering usual forensic routine.

## Experimental methods

### Chemicals and reagents

Water, methanol, acetonitrile, and acetone (all HPLC grade) were purchased from Honeywell (Seelze, Germany). Acetic acid and heptafluorobutyric acid (both purissima, p.a.) were obtained from Sigma-Aldrich (St. Louis, MO, USA). Reference standards and deuterated internal standards for THC, 11-OH-THC, THC-COOH, and CBD as well as a delta8-THC reference standard were purchased from Lipomed (Arlesheim, Switzerland). Reference standards of (9 R)- and (9S)-hexahydrocannabinol were obtained from Cayman Chemical (Ann Arbor, Michigan, USA). Validation procedures were performed using the (9 R)-hexahydrocannabinol standard (HHC).

Blank human plasma and whole-blood samples were obtained from the blood bank of the Innsbruck University hospital and were evaluated for absence of investigated analytes prior to use.

### Instrumentation

All experiments were performed on a Sciex 3200 QTRAP triple–quadrupole mass spectrometer equipped with a TurboV ESI source (Sciex, Foster City, CA, USA). The mass spectrometer was interfaced with a 1100 HPLC system (Agilent, Waldbronn, Germany) consisting of a 1100 pump, a 1100 four-channel degasser, and a 1100 column oven. An HTS PAL autosampler (CTC Analytics, Zwingen, Switzerland) equipped with a 20 µl injection loop was applied.

Sample evaporation under nitrogen was performed using an EVA EC1 evaporator (VLM, Bielefeld, Germany).

### Procedures

#### Preparation of standard solutions

A stock solution containing 500 µg/l of THC, 11-OH-THC, CBD, HHC, and 2000 µg/l THC-COOH, respectively, was prepared in methanol/water (75/25, v/v) and stored at −20°C. Dilutions of the stock solution with methanol/water (75/25, v/v) created calibrators at 0.5, 1.0, 2.5, 5.0, 10.0, 25.0 μg/l (THC, 11-OH-THC, CBD, HHC), and 2.0, 4.0, 10.0, 20.0, 40.0, 100 μg/l (THC-COOH), respectively, when fortifying 25 μl of standard solution into 250 μl of blank human plasma or whole blood. Quality control (QC) samples were prepared in methanol/water (75/25, v/v) from different vials than utilized for preparing standards. Low- and high QCs were prepared across the linear dynamic range of the assay at 1.0 µg/l and 10 µg/l (THC, 11-OH-THC, CBD, HHC) and 4.0 µg/l and 40 µg/l (THC-COOH), respectively.

Internal standard (IS) solution was prepared by diluting 100 µg/ml solutions of THC-d_3_, 11-OH-THC-d_3_, CBD-d_3_ and THC-COOH-d_3_ with methanol/water (75/25, v/v) resulting in concentrations of 0.1 µg/ml for THC-d_3_, 11-OH-THC-d_3_ and CBD-d_3_, and 0.4 µg/ml for THC-COOH-d_3_. IS solution was stored at −20°C and 25 μl of the solution was added to each 250 μl plasma or blood sample, providing final IS concentrations of 10 μg/l for THC-d_3_, 11-OH-THC-d_3_ and CBD-d_3_ and 40 µg/l for THC-COOH-d_3_. Since deuterated HHC was commercially not available during method development THC-d_3_ was used as IS for HHC quantification.

#### Sample preparation

Plasma or blood samples (250 µl) were mixed with 25 µl IS solution. For protein precipitation, 0.75 ml acetonitrile were added. The mixture was vortexed for 20 s and centrifuged for 5 min at 4000 × *g*. The supernatant was separated, diluted with 2 ml of a 0.1 M aqueous acetic acid solution and submitted to solid-phase extraction (SPE) employing Chromabond Drug II cartridges (45 µm, 200 mg, 3 ml, Macherey-Nagel, Dueren, Germany). The cartridges were equilibrated with 2 ml methanol and 2 ml water. After sample application, the cartridges were washed twice with 3 ml water each. To remove water, cartridges were placed in test tubes and centrifuged for 5 min at 4000 × *g*, and dried with nitrogen at 60°C for 20 min. Elution was accomplished with 2 ml acetone/acetic acid (99/1, v/v). The eluate was evaporated to dryness with nitrogen at 60°C and reconstituted in 35 µl of methanol/water (75/25, v/v).

#### Liquid chromatography

Chromatographic separation was accomplished on a Eurosphere II C18 column (100 × 2 mm, 5 μm, Knauer, Berlin, Germany) protected by a 0.2 µm stainless steel filter frit (Vici, Schenkon, Switzerland). The column oven temperature was 50°C throughout the analysis. The PAL autosampler was operated at room temperature, and the injection volume was 10 µl. Separations were performed using a 2 min gradient of 70–100% methanol in aqueous solution containing 0.5% acetic acid and 0.005% heptafluorobutyric acid (both v/v). The flow rate was set to 200 µl/min and the total run time was 15 min. The column outlet was directly connected to the electrospray ionization (ESI) source.

#### Mass spectrometry

Mass spectrometric data were acquired with ESI in positive ionization mode. MS–MS parameter settings (Table 1) were optimized via direct infusion of individual analytes (500 μg/l in initial mobile phase) at 10 µl/min. Optimized source parameters were as follows: spray voltage 5.5 kV, gas flows of 60 arbitrary units (gas 1 and gas 2) and 45 arbitrary units (curtain gas), respectively. Collision cell gas (CAD) was set to “medium” and the source temperature was adjusted to 500°C. Unit resolution was used for all experiments. Mass spectrometric data were recorded and analyzed on a personal computer with Analyst software 1.5 (Sciex).

**Table 1.** Experimental details for LC–MS–MS acquisition (bold font denotes quantifier transition)

Analyte	Retention time (min)	Q1 Mass (*m*/*z*)	Q3 Mass (*m*/*z*)	Dwell Time (ms)	DP (V)	EP (V)	CEP (V)	CE (V)	CXP (V)
THC	6.87	**315.0**	**193.4**	150	50	4.5	17.0	30	3.0
		315.0	123.2	50	55	3.7	25.0	44	4.0
THC-d_3_	6.86	318.3	196.1	50	50	4.5	17.0	30	3.0
11-OH-THC	5.94	**331.0**	**193.3**	500	45	4.0	20.0	34	7.9
		331.0	313.5	50	45	4.0	20.0	16	3.6
11-OH-THC-d_3_	5.92	334.0	316.5	200	45	4.0	20.0	16	3.6
HHC	7.06	**317.4**	**193.2**	50	50	4.5	17.0	30	3.0
		317.4	137.2	50	50	3.7	25.0	44	4.0
CBD	6.17	**315.0**	**193.4**	150	50	4.5	17.0	30	3.0
		315.0	123.2	50	55	3.7	25.0	44	4.0
CBD-d_3_	6.14	318.3	196.1	50	50	4.5	17.0	30	3.0
THC-COOH	6.05	**345.5**	**299.5**	50	60	5.0	13.3	30	5.1
		345.5	327.5	150	60	5.0	15.0	20	5.2
THC-COOH-d_3_	6.04	348.2	330.1	50	60	5.0	15.0	20	5.2

Abbreviations: Q1 = quadrupole 1, Q3 = quadrupole 3, DP = declustering potential, EP = entrance potential, CEP = collision cell entrance potential, CE = collision energy, CXP = collision cell exit potential).

#### Method validation

The method was fully validated for human plasma according to the guidelines of the German Society of Toxicological and Forensic Chemistry (GTFCh) [75]. Evaluated parameters included linearity, sensitivity, specificity, selectivity, intra- and inter-day precision, accuracy, carry-over, dilution integrity, matrix effects, extraction efficiency as well as autosampler and freeze–thaw stability. The applicability of the method to the analysis of whole-blood samples was evaluated and validated regarding selectivity, accuracy and precision, freeze–thaw stability, matrix effects, and extraction efficiency.

Linearity of the method was evaluated by analyzing seven replicates of spiked human plasma samples on seven different days at 0.5, 1.0, 2.5, 5.0, 10.0, 25.0 μg/l (THC, 11-OH-THC, CBD, HHC) and 2.0, 4.0, 10.0, 20.0, 40.0, 100 μg/l (THC-COOH), respectively. Peak area ratios of target analytes and their respective internal standards versus the nominated concentration of the levels were fitted in a nonweighted linear calibration model for all analytes. Linearity was expressed by the squared correlation coefficient (*R*^2^) and evaluated visually using residual plots.

Sensitivity was investigated by determining limits of detection (LOD) and limits of quantification (LOQ). LOD and LOQ were calculated from the linear calibration functions according to DIN 32 645 using the software SQS 2013 Version 1.00 for analytical method validation.

Endogenous interferences were assessed in six independent blank sources of human plasma and seven independent blank sources of human blood. Interferences were also assessed in blood and plasma fortified with internal standard. In addition, plasma was fortified with 80 illicit and common therapeutic drugs, metabolites, and related compounds including selected synthetic cannabinoids and analyzed for interfering signals (Supplementary Table S1). Specificity was based on retention time as well as on intensity ratios of two MRM transitions per analyte. Retention times for QC and authentic specimens were required to be within ±0.05 min of the mean calibrator retention time and the peak area ratios had to be within ±20% of the mean calibrator intensity ratio.

Accuracy and precision of the method were assessed by analyzing QC samples at low (low QC, corresponding LOQ) and high concentrations (high QC: 10 µg/l for THC, 11-OH-THC, CBD, HHC and 40 µg/l for THC-COOH). For plasma intra- and inter-day precision and accuracy were assessed by six determinations per concentration within one day and by nine determinations per concentration on nine different days whereas blood intra-day precision and accuracy was evaluated by seven determinations per concentration within one day. Precision was expressed as relative standard deviation (RSD %), which should be <15% at each concentration and <20% at LOQ level. Accuracy was expressed as a relative error (%), which was required to be within ±15% at each concentration and within ±20% at LOQ level.

Carryover was assessed in triplicate by analyzing negative blank matrix (internal standard only) after the highest calibrator. Carryover was considered insignificant if no peaks eluted at the analytes’ expected retention time (±0.05 min) and any signal present was less than LODs.

Dilution integrity of the method was assessed with three blank plasma specimens fortified with two-fold concentrations of the highest calibrator (50 and 200 µg/l for THC, 11-OH-THC, CBD, HHC and THC-COOH, respectively). Specimens were diluted with additional blank plasma at 1:5 ratios to yield a 250 μl volume. Samples and specimens were further processed as normal.

Extraction efficiency and matrix effects for each analyte were evaluated at two concentration levels (low QC and high QC) in six replicates according to the design of Matuszewski et al. [76] for plasma and blood. For determination of extraction efficiency, quality control standard solution was added prior to or following SPE. Extraction efficiency in percent was expressed as the mean analyte area of samples with control solution added before SPE (*n* = 6) divided by the mean analyte area of samples with control solution added after SPE (*n* = 6). Matrix effect was investigated by comparing analyte peak areas of extracted blank samples that were fortified after SPE versus analyte peak areas of neat samples prepared in methanol/water (75/25, v/v) at equivalent concentrations and was calculated by dividing the analyte areas of blank samples fortified after SPE by areas of neat samples, expressed as percent.

Processed sample stability for plasma samples while stored in the autosampler at room temperature was evaluated. Ten replicates of extracted low and high QC samples were analyzed repeatedly after ∼4 h resulting in an overall investigated time frame of 41.5 h. Processed samples were considered stable if peak area loss was <15%. Freeze-thaw analyte stability was evaluated for plasma and whole blood at low QC and high QC concentrations with six replicates each, prior and after three freeze–thaw cycles (one cycle corresponds to 20 h freeze and 1 h thaw at room temperature). All samples were quantified from the calibration curve initially analyzed.

## Results and discussion

### Method development

The aim of the current work was to establish and validate an efficient and robust LC–MS–MS method based on the available low-end instrumentation enabling the simultaneous determination of THC, 11-OH-THC, THC-COOH, HHC, and CBD in human plasma and blood. Main requirements of the newly developed method were better sensitivity and higher sample throughput in combination with less hands-on time as compared to the previously available GC–MS method. Another key prerequisite was the unambiguous and reliable differentiation between “traditional” cannabis consumption and consumption of HHC and/or CBD products. Essentially, artificial transformation of CBD to THC, which can be observed in GC–MS workflows depending on the conditions of sample preparation, derivatization and/or GC–MS analysis, had to be eliminated.

**Figure 1.**
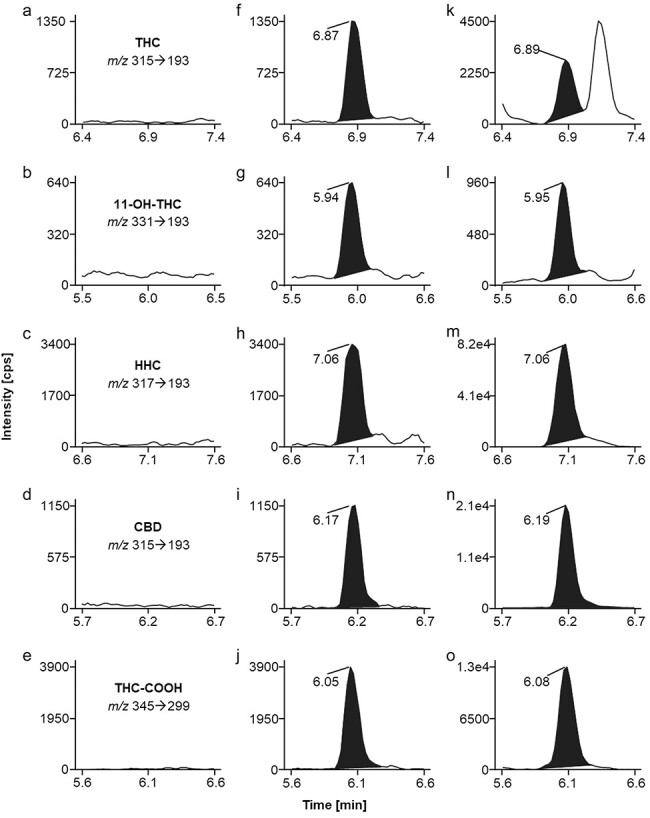
MRM ion chromatograms of (a–e) extracted blank plasma, (f–j) analytes at limit of quantification (LOQ: THC, 11-OH-THC, HHC, CBD 1.0 µg/l; THC-COOH 4.0 µg/l), and (k–o) a DUID casework plasma sample containing all target analytes.

MS–MS conditions were optimized by direct infusion of solutions of each individual analyte and internal standard. Source parameters are detailed in the “Experimental methods” section and MRM parameters are listed in Table 1. Two MRM transitions for each analyte were monitored for unambiguous identification. For quantification the highlighted (bold font) MRM transition (see Table 1) was used. Notably, identical MRM conditions are mentioned for THC and CBD as well as for the respective internal standards. THC and CBD are structurally related compounds, with CBD being considered the open-chain form and THC the related cyclic form. The two compounds exhibit a high degree of similarity of fragment ion mass spectra, which leads to interference in the MRM traces. As the presented chromatographic method clearly differentiates the two cannabinoids with CBD eluting 0.60 min earlier than THC (Fig. 1f and i), identical MRM settings can be applied for monitoring both, THC and CBD as well as their deuterated analogs. Thereby mass spectrometric detection is taking advantage of “saving” cycle time which is beneficial using an instrument with limited scan speed.

Optimization of chromatographic conditions was restricted because other accredited routine applications were performed on the same LC–MS–MS system as well. Applying the established reversed phase column and mobile phases enabled the separation of target analytes within a runtime of 15 min with exception of the two HHC diastereomers. 9 R- and 9S-HHC eluted at identical retention times regardless of the applied methanol gradient, column temperature and flow rate. Hence, the procedure determines “total HHC concentrations” in biological samples, not distinguishing 9 R- and 9S-HHC. Likewise, the present chromatographic system enabled only partial separation of delta9- and delta8-THC. Delta8-THC eluted 0.1 min later than delta9-THC but baseline separation of the two isomers was not achieved. Therefore, delta8-THC was omitted from the validated method. However, for casework analysis misidentification of the two isomers can be excluded due to a retention time difference larger than ±0.05 min, which is the chromatographic identification criterion for all analytes included in the validated method.

As solvent mixture for reconstitution of extracts methanol/water (75/25, v/v) was used, as higher methanol percentage impaired chromatographic behavior and lower methanol content reduced solubility of analytes. The injection volume of 10 µl was a compromise between signal intensity and saving of extract volume for potentially needed repeating injections.

In the course of method development different sample preparation techniques were evaluated including liquid–liquid extraction, supported liquid extraction, protein precipitation as well as solid-phase extraction. Attempts of performing one-step sample preparation compromised analytical performance of the method. Finally, the presented elaborate workflow including protein precipitation and consecutive solid-phase extraction generates clean extracts showing little endogenous interferences. Low noise in MRM traces combined with reasonable extraction efficiency enabled sensitive and selective detection of all analytes even at low concentrations with low sample volume input on the available low-end instrumentation.

### Method validation

The method was validated according to the criteria described in the “Experimental” section. Calibration curves (Table 2) showed excellent linearity (*R*^2^ > 0.999, no weighting) over the concentration range of 0.5–25 µg/l for THC, 11-OH-THC, HHC, and CBD, and 2.0–100 µg/l for THC-COOH, respectively. Visual evaluation of calibrations using residual plots suggested that linear calibration models were appropriate as individual residuals showed equal variances across the calibration range and random distribution around the zero line. Back-calculated concentrations were all within 85%–115% of the nominal concentrations.

**Table 2.** LOD, LOQ, and calibration results for THC, 11-OH-THC, HHC, CBD, and THC-COOH in human plasma

Analyte	Internal standard	LOD (µg/l)	LOQ (µg/l)	Slope ± SD (*n* = 7)	*y*-int ± SD (*n* = 7)	*R* ^2^ ± SD (*n* = 7)	Linear range (µg/l)
THC	THC-d_3_	0.13	0.65	0.065 ± 0.001	0.005 ± 0.009	0.999 ± 0.001	0.5–25
11-OH-THC	11-OH-THC-d_3_	0.04	0.20	0.036 ± 0.001	0.002 ± 0.001	1.000 ± 0.000	0.5–25
HHC	THC-d_3_	0.13	0.67	0.280 ± 0.004	0.020 ± 0.004	0.999 ± 0.001	0.5–25
CBD	CBD-d_3_	0.07	0.36	0.061 ± 0.001	0.002 ± 0.005	0.999 ± 0.001	0.5–25
THC-COOH	THC-COOH-d_3_	0.72	3.61	0.023 ± 0.001	0.002 ± 0.018	0.999 ± 0.000	2.0–100

SD = standard deviation; *y*-int = *y*-intercept.

Statistically determined values for LOD and LOQ (Table 2) were clearly below 0.5 and 1.0 µg/l (THC, 11-OH-THC, HHC, CBD) and 2.0 and 4.0 µg/l (THC-COOH) which are comparable to recently published methods [71–74] and applicable limits in forensic caseworks, fulfilling the requirements regarding sensitivity.

No interfering signals were detected for all analytes in six independent sources of plasma. Figure 1(a–e) depicts representative clean MRM chromatograms of an extracted blank plasma sample, proving selectivity of the method. Apparently, potentially interfering endogenous species are effectively removed during the applied sample preparation steps. Furthermore, no chromatographic peaks were observed for plasma samples fortified with 80 potentially interfering exogenous substances (Supplementary Table S1). The exception was the naturally occurring cannabinoid cannabigerol that is structurally related to HHC, with cannabigerol being considered the open-chain form and HHC the related cyclic form. These two compounds exhibit a high degree of similarity of fragment ion mass spectra, which may lead to interference in the MRM traces. However, the presented chromatographic method clearly differentiates the two cannabinoids with cannabigerol eluting 0.90 min earlier than HHC.

Possible carryover in a negative blank matrix following a specimen spiked at the upper limit of quantification level was assessed. Any signal present was less than each LOD, suggesting that the current method application was devoid of carryover.

In the course of method validation, the required identification criteria were met for all analytes.

Table 3 shows accuracy and precision data evaluated for each analyte at two concentration levels in human plasma. The intra-day accuracy, calculated as the relative error of target concentrations, ranged from −7.0% to 5.6%. The maximum intra-day precision (% RSD) was 13.0%. The inter-day accuracy ranged from −11.8% to 11.3%. Inter-day precision (% RSD) was ≤12.7%.

**Table 3.** Precision and accuracy data in human plasma at two concentration levels of 1.0 and 10.0 µg/l (THC, 11-OH-THC, HHC, CBD) as well as 4.0 and 40 µg/l (THC-COOH)

	Intra-day (*n* = 6)				Inter-day (*n* = 9)			
	Precision (% RSD)		Accuracy (rel. error, %)		Precision (% RSD)		Accuracy (rel. error, %)	
Analyte	Low	High	Low	High	Low	High	Low	High
THC	5.1	7.3	1.0	−6.8	11.5	7.3	−5.0	−7.5
11-OH-THC	9.4	9.1	4.0	5.2	12.7	7.6	7.0	8.0
HHC	11.6	13.0	1.0	5.6	11.6	4.8	1.0	−4.0
CBD	6.1	11.9	1.0	−1.4	12.6	6.2	−1.0	−11.8
THC-COOH	8.6	8.6	−7.0	0.8	9.2	11.0	−7.8	11.3

RSD = relative standard deviation.

Extraction efficiency and matrix effect data for plasma samples are presented in Table 4. The mean extraction efficiency of target analytes ranged from 73.8% to 82.6%, suggesting that the developed sample preparation procedure had good extraction efficiency. The matrix effect at two concentration levels was in the range of 76.0%–92.6%, which suggested that co-eluting substances had only minor effects on the ionization of target analytes.

**Table 4.** Extraction efficiency and matrix effects in human plasma at two concentration levels of 1.0 and 10.0 µg/l (THC, 11-OH-THC, HHC, CBD) as well as 4.0 and 40 µg/l (THC-COOH)

	Extraction efficiency (%, *n* = 6)		Matrix effect (%, *n* = 6)	
Analyte	Low	High	Low	High
THC	77.2	79.1	77.4	77.3
11-OH-THC	78.1	81.3	77.4	76.0
HHC	73.8	74.4	84.7	85.2
CBD	79.1	79.6	81.5	78.6
THC-COOH	75.3	82.6	87.2	92.6

RSD = relative standard deviation.

Accuracy, precision data, extraction efficiency, and matrix effect data were comparable to published data [71–74, 77].

Dilution integrity was maintained up to five times dilution with blank plasma and all analytes were quantified within 20% of the theoretical concentration. This proved that high concentrations of target analytes that are occasionally observed in casework samples following cannabis smoking can be reliably measured.

Stability of extracted plasma specimens at room temperature on the autosampler was determined for 41.5 h. All analytes were stable under these conditions over the investigated time frame as there was no significant decrease (<15%) of peak areas observed. In fortified plasma samples at low and high QC concentrations (six replicates), all analytes were stable during three freeze–thaw cycles.

### Applicability to whole-blood samples

Separation of plasma is not always possible due to hemolysis especially in postmortem blood samples, or due to low sample volume. This means that a significant number of whole-blood samples are regularly received in forensic laboratories. Therefore, one essential aspect during validation was the applicability of the method to blood samples. Seven independent sources of whole-blood samples were extracted and analyzed in order to assess selectivity. No interferences were detected. Potentially interfering endogenous species were obviously effectively removed during the applied sample preparation steps.

In order to assess the applicability of matrix-matched plasma calibrations for analyte quantitation, low and high QC specimens were generated in seven independent blood samples and analyzed. Quantitative results for each analyte were determined applying the respective plasma calibration, and accuracy and precision were calculated for both concentration levels (Table 5). Relative errors were within −15% to 4.2% for all analytes and precision (% RSD) was less than 8.3%. These results prove that analysis of blood samples by applying matrix matched plasma calibrations was valid.

**Table 5.** Extraction efficiency, matrix effects, intra-day precision, and accuracy data in human whole blood at two concentration levels of 1.0 and 10.0 µg/l (THC, 11-OH-THC, HHC, CBD) as well as 4.0 and 40 µg/l (THC-COOH)

	Extraction efficiency (%, *n* = 6)		Matrix effect (%, *n* = 6)		Precision (% RSD, *n* = 7)		Accuracy (rel. error, %, *n* = 7)	
Analyte	Low	High	Low	High	Low	High	Low	High
THC	72.2	81.9	98.6	93.7	5.5	1.4	−3.0	−8.0
11-OH-THC	72.5	78.2	80.4	83.1	3.5	2.9	4.0	13.0
HHC	62.0	73.2	115.2	106.3	8.3	5.8	−11.0	4.0
CBD	71.9	84.4	94.8	85.5	6.1	4.4	0.0	−15.0
THC-COOH	62.9	79.7	105.1	100.6	5.5	6.6	2.0	4.2

Mean extraction efficiency and matrix effect data for blood samples are also presented in Table 5. Mean extraction efficiency of target analytes ranged from 62.0% to 84.4% and show that the applied sample preparation procedure has acceptable extraction efficiency also for whole-blood samples. Matrix effects were in the range of 80.4% to 115.2%, which suggests that co-eluting substances have only minor to moderate effects on the ionisation of target analytes.

In fortified whole blood at low and high QC concentrations (six replicates), all analytes were stable during three freeze–thaw cycles. Average concentrations of stability samples were within 90–110% of the respective control samples.

Furthermore, applicability of the method to blood samples was demonstrated by regular successful participation in proficiency tests.

### Limitations of the method

The application of deuterated HHC as internal standard for HHC quantitation would be beneficial for compensation of matrix effects and extraction efficiency fluctuations, especially for the analysis of blood samples (Table 5). During method development and validation no deuterated analogues of HHC had been commercially available. Meanwhile, this has changed and the inclusion of HHC-d_9_ to the presented method is scheduled.

The presented method determines total HHC concentrations in biological samples. This analytical limitation could be overcome by applying an alternative reversed phase chromatographic system [71] or by applying chiral chromatography [72]. Currently, no differentiation between the two HHC diastereomers is stated in the Austrian legislation regarding legal consequences for the driver [61].

At the current stage of development, delta8-THC is not included in the validated method due to insufficient chromatographic separation from delta9-THC. Although baseline separation is not achieved, the chromatographic method distinguishes reliably the two isomers due to differing retention times.

The method covers metabolites of THC but not of CBD and HHC. Monitoring metabolites of these two targets would further improve the method. For the respective carboxy-metabolites, higher concentrations and/or longer detection windows compared to their precursors are observed in biological samples [71, 78]. Thereby carboxy-metabolites of HHC and CBD can be considered as appropriate markers for proving the intake of the respective cannabinoid. Additionally, chronic consumption can be differentiated from single-dose users according to levels of carboxy-metabolites, which can be important information in forensic casework. Applying the present method in CBD positive samples putative signals of CBD-COOH were detected in the MRM traces of THC-COOH due to a high degree of similarity of fragment ion mass spectra. Again, the two analytes were unequivocally differentiated by chromatography, with CBD-COOH eluting 2.5 min earlier than THC-COOH.

### Casework analysis

Application to casework samples proved the robustness and capacity of the method in a forensic routine laboratory. It was fully accredited according to EN ISO/IEC 17 025. Since its implementation in routine work about one thousand DUID cases were analyzed until the end of 2023. The majority of samples, about 80%, was tested positive for THC and/or its main metabolites, with THC concentrations above LOQ ranging from 1.0 to 200 µg/l (mean 8.1 µg/l). In Austria, no threshold values for illicit drugs are defined in the road traffic act. This means that the presence of a substance in blood, independent of its concentration, leads to legal consequences for the driver. Having this in mind, the applied LOQs of the presented method, namely 1.0 µg/l for the psychoactive cannabinoids, is reasonable and in good accordance with international THC limits such as the commonly applied analytical limit of 1.0 µg/l in Germany [79] or 1.5 µg/l in Switzerland [80].

A small number of DUID samples was tested positive for CBD (*n* = 33) with concentrations ranging from 1.0 to 27.0 µg/l. Measured levels above 5.0 µg/l are concordant with concentrations that were observed within 1–2 h after smoking CBD joints [81]. The majority of these samples were concomitantly tested positive for THC, indicating that an exclusive consumption of pure CBD products is rare, at least among DUID cases.

Even fewer samples contained HHC (*n* = 6). Obviously, HHC has lost importance at least in Austria. It can be only speculated if the reason for this may be truly found in the Austrian HHC regulation by law [61]. Measured plasma concentrations ranged from 2.1 to 37.0 µg/l. HHC plasma levels seem to be similar to THC levels, and the observed HHC concentrations are in good accordance with published data [71–73]. It can be reasonably assumed that the highest measured value of 37.0 µg/l is observed shortly after consumption, especially in regard to measured levels of about 4.0 µg/l (9 R + 9S) HHC in a user stating regular consumption of HHC products [74]. In all of the six HHC positive cases, plasma as well as blood samples were available and consequently analyzed. Measured blood levels ranged from 0.6 to 9.1 µg/l and a blood to plasma (b/p) ratio of 0.28 (0.24–0.39) was determined for HHC, which is about half the THC b/p ratio of 0.5–0.6 [82].

Figure 1(k–o) shows MRM ion chromatograms of a DUID casework plasma sample that contained all analytes covered by the presented method. The obtained concentrations were 2.3 µg/l THC, 1.3 µg/l 11-OH-THC, 37.0 µg/l HHC, 22.0 µg/l CBD, and 14.3 µg/l THC-COOH, respectively. Apparently, the three cannabinoids THC, HHC as well as CBD had been consumed. Concerning the observed plasma levels the user had potentially intended to take an HHC and/or CBD containing product, which was contaminated with THC. However, no further information about consuming habits and/or time range between vehicle control and blood sampling were available.

The presented method was additionally applied to postmortem blood and plasma samples. The analysis of such samples can be challenging, due to matrix effects dependent on the time interval between time of death and blood sampling. Sample preparation was successfully performed according to standard procedure. Target analytes were detected, and no interference with putrefaction products was observed. However, in none of the examined postmortem cases HHC and CBD were present.

## Conclusion

A fully validated, sensitive, and specific analytical LC–MS–MS method for simultaneous determination of THC, 11-OH-THC, THC-COOH, HHC, and CBD in human blood and plasma samples is presented. The method is easy to handle, requires reasonable sample volumes and does not need high-end analytical equipment. Since its implementation and accreditation according to ISO 17 025, the method has proven to be robust and fit for purpose in long-term routine analysis not only in DUID cases but also with postmortem samples. Furthermore, the design of the method allows for an easy and uncomplicated extension to further cannabinoids and/or their main metabolites, if required, thus being prepared for new challenges within a quickly changing cannabis market including semisynthetic cannabinoids.

## Supplementary Material

bkae094_Supp

## Data Availability

The data underlying the validation results in this article will be shared upon reasonable request to the corresponding author. Casework data cannot be shared for ethical/privacy reasons.

## References

[1] Adams R . Marihuana. *Science* 1940;92:115–19. doi: 10.1126/science.92.2380.115 17730206

[2] Adams R . Marihuana: Harvey Lecture, February 19, 1942. *Bull N Y Acad Med* 1942;18:705–30. 19312292 PMC1933888

[3] Turner CE, Elsohly MA, Boeren EG. Constituents of Cannabis sativa L. XVII. A review of the natural constituents. *J Nat Prod* 1980;43:169–234. doi: 10.1021/np50008a001 6991645

[4] Hua T, Vemuri K, Nikas SP et al. Crystal structures of agonist-bound human cannabinoid receptor CB(1). *Nature* 2017;547:468–71.doi: 10.1038/nature23272 28678776 PMC5793864

[5] Taura F, Sirikantaramas S, Shoyama Y et al. Phytocannabinoids in Cannabis sativa: recent studies on biosynthetic enzymes. *Chem Biodivers* 2007;4:1649–63.doi: 10.1002/cbdv.200790145 17712812

[6] Matsuda LA, Lolait SJ, Brownstein MJ et al. Structure of a cannabinoid receptor and functional expression of the cloned cDNA. *Nature* 1990;346:561–64.doi: 10.1038/346561a0 2165569

[7] Herkenham M, Lynn AB, Little MD et al. Cannabinoid receptor localization in brain. *Proc Natl Acad Sci USA* 1990;87:1932–36.doi: 10.1073/pnas.87.5.1932 2308954 PMC53598

[8] Wenger T, Ledent C, Csernus V et al. The central cannabinoid receptor inactivation suppresses endocrine reproductive functions. *Biochem Biophys Res Commun* 2001;284:363–68.doi: 10.1006/bbrc.2001.4977 11394887

[9] Puhl SL . Cannabinoid-sensitive receptors in cardiac physiology and ischaemia. *Biochim Biophys Acta Mol Cell Res* 2020;1867:118462. doi: 10.1016/j.bbamcr.2019.03.009 30890410

[10] Mendizabal-Zubiaga J, Melser S, Bénard G et al. Cannabinoid CB(1) receptors are localized in striated muscle mitochondria and regulate mitochondrial respiration. *Front Physiol* 2016;7:476.doi: 10.3389/fphys.2016.00476 PMC5078489 27826249

[11] McLaughlin PJ, Thakur GA, Vemuri VK et al. Behavioral effects of the novel potent cannabinoid CB1 agonist AM 4054. *Pharmacol Biochem Behav* 2013;109:16–22.doi: 10.1016/j.pbb.2013.04.011 23603029 PMC4015344

[12] Axelrod J, Felder CC. Cannabinoid receptors and their endogenous agonist, anandamide. *Neurochem Res* 1998;23:575–81. doi: 10.1023/A:1022418217479 9566594

[13] Gaoni Y, Isolation MR. Structure, and partial synthesis of an active constituent of hashish. *J Am Chem Soc* 1964;86:1646–47. doi: 10.1021/ja01062a046

[14] Makriyannis A . 2012 Division of medicinal chemistry award address. Trekking the cannabinoid road: a personal perspective. *J Med Chem* 2014;57:3891–911. doi: 10.1021/jm500220s 24707904 PMC4064474

[15] Mechoulam R, Hanuš LO, Pertwee R et al. Early phytocannabinoid chemistry to endocannabinoids and beyond. *Nat Rev Neurosci* 2014;15:757–64.doi: 10.1038/nrn3811 25315390

[16] Munro S, Thomas KL, Abu-Shaar M. Molecular characterization of a peripheral receptor for cannabinoids. *Nature* 1993;365:61–65. doi: 10.1038/365061a0 7689702

[17] Felder CC, Joyce KE, Briley EM et al. Comparison of the pharmacology and signal transduction of the human cannabinoid CB1 and CB2 receptors. *Mol Pharmacol* 1995;48:443–50. 7565624

[18] Fulmer ML, Thewke DP. The endocannabinoid system and heart disease: the role of cannabinoid receptor type 2. *Cardiovasc Hematol Disord Drug Targets* 2018;18:34–51. doi: 10.2174/1871529X18666180206161457 29412125 PMC6020134

[19] Howlett AC, Barth F, Bonner TI et al. International Union of Pharmacology. XXVII. Classification of cannabinoid receptors. *Pharmacol Rev* 2002;54:161–202.doi: 10.1124/pr.54.2.161 12037135

[20] Rakotoarivelo V, Mayer TZ, Simard M et al. The Impact of the CB(2) Cannabinoid Receptor in Inflammatory Diseases: An Update. *Molecules* 2024;29:3381.doi: 10.3390/molecules29143381 PMC11279428 39064959

[21] Yang W, Gong X, Sun H et al. Discovery of a CB(2) and 5-HT(1A) receptor dual agonist for the treatment of depression and anxiety. *Eur J Med Chem* 2024;265:116048.doi: 10.1016/j.ejmech.2023.116048 38150961

[22] Bartkowiak-Wieczorek J, Bienert A, Czora-Poczwardowska K et al. Cannabis sativa L. extract alleviates neuropathic pain and modulates CB1 and CB2 receptor expression in rat. *Biomolecules* 2024;14:1065.doi: 10.3390/biom14091065 PMC11430414 39334832

[23] Carruthers ER, Grimsey NL. Cannabinoid CB(2) receptor orthologues; in vitro function and perspectives for preclinical to clinical translation. *Br J Pharmacol* 2024;181:2247–69. doi: 10.1111/bph.16172 37349984

[24] Vučković S, Srebro D, Vujović KS. Cannabinoids and pain: new insights from old molecules. *Front Pharmacol* 2018;9:1259. doi: 10.3389/fphar.2018.01259 PMC6277878 30542280

[25] Velasco G, Hernández-Tiedra S, Dávila D et al.. The use of cannabinoids as anticancer agents. *Prog Neuropsychopharmacol Biol Psychiatry* 2016;64:259–66.doi: 10.1016/j.pnpbp.2015.05.010 26071989

[26] Kelly P, Jones RT. Metabolism of tetrahydrocannabinol in frequent and infrequent marijuana users. *J Anal Toxicol* 1992;16:228–35. doi: 10.1093/jat/16.4.228 1323733

[27] Huestis MA, Henningfield JE, Cone EJ. Blood cannabinoids. I. Absorption of THC and formation of 11-OH-THC and THCCOOH during and after smoking marijuana. *J Anal Toxicol* 1992;16:276–82. doi: 10.1093/jat/16.5.276 1338215

[28] Toennes SW, Ramaekers JG, Theunissen EL et al. Comparison of cannabinoid pharmacokinetic properties in occasional and heavy users smoking a marijuana or placebo joint. *J Anal Toxicol* 2008;32:470–77.doi: 10.1093/jat/32.7.470 18713514

[29] Wall ME, Sadler BM, Brine D et al. Metabolism, disposition, and kinetics of delta-9-tetrahydrocannabinol in men and women. *Clin Pharmacol Ther* 1983;34:352–63.doi: 10.1038/clpt.1983.179 6309462

[30] Manno JE, Manno BR, Kemp PM et al. Temporal indication of marijuana use can be estimated from plasma and urine concentrations of delta9-tetrahydrocannabinol, 11-hydroxy-delta9-tetrahydrocannabinol, and 11-nor-delta9-tetrahydrocannabinol-9-carboxylic acid. *J Anal Toxicol* 2001;25:538–49.doi: 10.1093/jat/25.7.538 11599597

[31] Agurell S, Halldin M, Lindgren JE et al. Pharmacokinetics and metabolism of delta 1-tetrahydrocannabinol and other cannabinoids with emphasis on man. *Pharmacol Rev* 1986;38:21–43. 3012605

[32] Johansson E, Halldin MM. Urinary excretion half-life of delta 1-tetrahydrocannabinol-7-oic acid in heavy marijuana users after smoking. *J Anal Toxicol* 1989;13:218–23. doi: 10.1093/jat/13.4.218 2550702

[33] McBurney LJ, Bobbie BA, Sepp LA. GC/MS and EMIT analyses for delta 9-tetrahydrocannabinol metabolites in plasma and urine of human subjects. *J Anal Toxicol* 1986;10:56–64. doi: 10.1093/jat/10.2.56 3009969

[34] Lemberger L, Axelrod J, Kopin IJ. Metabolism and disposition of delta-9-tetrahydrocannabinol in man. *Pharmacol Rev* 1971;23:371–80. 4943951

[35] Chayasirisobhon S . Mechanisms of action and pharmacokinetics of Cannabis. *Perm J* 2020;25:200. 10.7812/TPP/19.200 PMC8803256 33635755

[36] Grotenhermen F . Pharmacokinetics and pharmacodynamics of cannabinoids. *Clin Pharmacokinet* 2003;42:327–60. doi: 10.2165/00003088-200342040-00003 12648025

[37] Ohlsson A, Lindgren JE, Wahlen A et al. Plasma delta-9 tetrahydrocannabinol concentrations and clinical effects after oral and intravenous administration and smoking. *Clin Pharmacol Ther* 1980;28:409–16.doi: 10.1038/clpt.1980.181 6250760

[38] Thomas A, Baillie GL, Phillips AM et al. Cannabidiol displays unexpectedly high potency as an antagonist of CB1 and CB2 receptor agonists in vitro. *Br J Pharmacol* 2007;150:613–23.doi: 10.1038/sj.bjp.0707133 17245363 PMC2189767

[39] Campos AC, Moreira FA, Gomes FV et al. Multiple mechanisms involved in the large-spectrum therapeutic potential of cannabidiol in psychiatric disorders. *Philos Trans R Soc Lond B Biol Sci* 2012;367:3364–78.doi: 10.1098/rstb.2011.0389 23108553 PMC3481531

[40] European Union law, ECLI:EU:C:2020:938 . Reference for a preliminary ruling – Free movement of goods – Common organisation of the markets in the flax and hemp sector – Exceptions – Protection of public health – National legislation limiting the industrialisation and marketing of hemp solely to fibre and seeds – Cannabidiol (CBD). (2020). https://eur-lex.europa.euhttps://eur-lex.europa.eu/legal-content/EN/TXT/PDF/?uri=CELEX:62018CJ0663&qid=1720330299826 (14 June 2024, date last accessed).

[41] Hill AJ, Williams CM, Whalley BJ et al. Phytocannabinoids as novel therapeutic agents in CNS disorders. *Pharmacol Ther* 2012;133:79–97.doi: 10.1016/j.pharmthera.2011.09.002 21924288

[42] Union Register of medicinal products for human use (2024) . Epidyolex. https://ec.europa.eu/health/documents/community-register/html/h1389.htm (14 June 2024, date last accessed).

[43] Chico SFV, Diaz DAM, Contreras-Puentes N. Use of cannabidiol in the treatment of drug-refractory epilepsy in children and young adults: A systematic review. *J Neurosci Rural Pract* 2024;15:203–10. doi: 10.25259/JNRP_618_2023 38746511 PMC11090527

[44] Carus M, European Industrial Hemp Association. Position paper of the European Industrial Hemp Association (EIHA) on Reasonable regulation of cannabidiol (CBD) in food, cosmetics, as herbal natural medicine and as medicinal product. 2018. https://eiha.org/media/2016/10/18-10-EIHA-CBD-position-paper.pdf (14 June 2024, date last accessed).

[45] Huesti MA, Solimini R, Pichini S et al. Cannabidiol adverse effects and toxicity. *Curr Neuropharmacol* 2019;17:974–89.doi: 10.2174/1570159X17666190603171901 31161980 PMC7052834

[46] Madeo G, Kapoor A, Giorgetti R et al. Update on cannabidiol clinical toxicity and adverse effects: a systematic review. *Curr Neuropharmacol* 2023;21:2323–42.doi: 10.2174/1570159X21666230322143401 36946485 PMC10556379

[47] European Union Drugs Agency (EUDA) . EMCDDA report: Low-THC cannabis products in Europe. 2020. https://www.euda.europa.eu/publications/ad-hoc-publication/low-thc-cannabis-products-europe_en (07 July 2024, date last accessed).

[48] Statista . CBD Produkte - Oesterreich. https://de.statista.com/outlook/hmo/cannabis/cbd-produkte/oesterreich (08 October 2024, date last accessed).

[49] Nikas SP, Alapafuja SO, Papanastasiou I et al. Novel 1ʹ,1ʹ-chain substituted hexahydrocannabinols: 9β-hydroxy-3-(1-hexyl-cyclobut-1-yl)-hexahydrocannabinol (AM2389) a highly potent cannabinoid receptor 1 (CB1) agonist. *J Med Chem* 2010;53:6996–7010.doi: 10.1021/jm100641g 20925434 PMC3650853

[50] Järbe TU, Tai S, LeMay BJ et al. AM2389, a high-affinity, in vivo potent CB1-receptor-selective cannabinergic ligand as evidenced by drug discrimination in rats and hypothermia testing in mice. *Psychopharmacology* 2012;220:417–26.doi: 10.1007/s00213-011-2491-1 21989802 PMC3291515

[51] Papanastasiou IP, Georgiadis MO, Iliopoulos-Tsoutsouvas C et al. Improved cyclobutyl nabilone analogs as potent CB1 receptor agonists. *Eur J Med Chem* 2022;230:114027.doi: 10.1016/j.ejmech.2021.114027 35051750

[52] Nye JS, Seltzman HH, Pitt CG et al. High-affinity cannabinoid binding sites in brain membranes labeled with [3H]-5ʹ-trimethylammonium delta 8-tetrahydrocannabinol. *J Pharmacol Exp Ther* 1985;234:784–91. 2993595

[53] European Union Drugs Agency (EUDA) Ujvary I . EMCDDA Technical Report, Hexahydrocannabinol (HHC) and related substances. (2023). https://www.euda.europa.eu/publications/technical-reports/hhc-and-related-substances_en (07 July 2024, date last accessed).

[54] Casati S, Rota P, Bergamaschi RF et al. Hexahydrocannabinol on the light cannabis market: the latest “New” Entry. *Cannabis Cannabinoid Res* 2024;9:622–28.doi: 10.1089/can.2022.0253 36445181

[55] Meehan-Atrash J, Rahman I. Cannabis vaping: existing and emerging modalities, chemistry, and pulmonary toxicology. *Chem Res Toxicol* 2021;34:2169–79. doi: 10.1021/acs.chemrestox.1c00290 34622654 PMC8882064

[56] Collins A, Ramirez G, Tesfatsion T et al. Synthesis and characterization of the diastereomers of HHC and H4CBD. *Nat Prod Commun* 2023;18:1934578X231158910. doi: 10.1177/1934578X231158910

[57] Marzullo P, Foschi F, Coppini DA et al. Cannabidiol as the substrate in acid-catalyzed intramolecular cyclization. *J Nat Prod* 2020;83:2894–901.doi: 10.1021/acs.jnatprod.0c00436 32991167 PMC8011986

[58] Reggio PH, Greer KV, Cox SM. The importance of the orientation of the C9 substituent to cannabinoid activity. *J Med Chem* 1989;32:1630–35. doi: 10.1021/jm00127a038 2738895

[59] Archer RA, Boyd DB, Demarco PV et al. Structural studies of cannabinoids. A theoretical and proton magnetic resonance analysis. *J Am Chem Soc* 1970;92:5200–06.doi: 10.1021/ja00720a033 5432664

[60] Mechoulam R, Lander N, Varkony TH et al. Stereochemical requirements for cannabinoid activity. *J Med Chem* 1980;23:1068–72.doi: 10.1021/jm00184a002 7420350

[61] European Legislation Identifier (ELI) . Änderung der Neue-Psychoaktive-Substanzen-Verordnung. (2023). https://www.ris.bka.gv.at/eli/bgbl/II/2023/73/20230322 (14 June 2024, date last accessed).

[62] European Legislation Identifier (ELI) . Verordnung des EDI über die Verzeichnisse der Betäubungsmittel, psychotropen Stoffe, Vorläuferstoffe und Hilfschemikalien. (2023). https://www.fedlex.admin.ch/eli/oc/2023/173/de (14 June 2024, date last accessed).

[63] Hartman RL, Huestis MA. Cannabis effects on driving skills. *Clin Chem* 2013;59:478–92. doi: 10.1373/clinchem.2012.194381 23220273 PMC3836260

[64] Desrosiers NA, Ramaekers JG, Chauchard E et al. Smoked cannabis’ psychomotor and neurocognitive effects in occasional and frequent smokers. *J Anal Toxicol* 2015;39:251–61.doi: 10.1093/jat/bkv012 25745105 PMC4416120

[65] National Highway Traffic Safety Administration . Drug and Alcohol Crash Risk: A Case Control Study. (2016). https://www.nhtsa.gov/behavioral-research/drug-and-alcohol-crash-risk-study (14 June 2024, date last accessed).

[66] Gelmi TJ, Weinmann W, Pfäffli M. Impact of smoking cannabidiol (CBD)-rich marijuana on driving ability. *Forensic Sci Res* 2021;6:195–207. doi: 10.1080/20961790.2021.1946924 34868711 PMC8635612

[67] United States Department of Transportation . Update to Special Reports on Traffic Safety during the COVID-19 Public Health Emergency: Fourth Quarter Data. 2021. 10.21949/1526015 (14 June 2024, date last accessed).

[68] Kuratorium fuer Verkehrssicherheit Oesterreich . *Dunkelfeldstudie Drogen*. 2023. https://www.kfv.at/kfv-dunkelfeldstudie-drogen-am-steuer-detektiert-erneuten-anstieg/ (14 June 2024, date last accessed).

[69] Myers MG, Bonar EE, Bohnert KM. Driving under the influence of cannabis, alcohol, and illicit drugs among adults in the United States from 2016 to 2020. *Addict Behav* 2023;140:107614. doi: 10.1016/j.addbeh.2023.107614 36652810

[70] Willeman T, Bartolli M, Jourdil JF et al.. Trends in drivers testing positive for drugs of abuse in oral fluid from 2018 to 2021 in France. *Forensic Sci Int* 2023;352:111835.doi: 10.1016/j.forsciint.2023.111835 37748427

[71] Kronstrand R, Roman M, Green H et al. Quantitation of hexahydrocannabinol (HHC) and metabolites in blood from DUID cases. *J Anal Toxicol* 2024;48:235–41.doi: 10.1093/jat/bkae030 38581662

[72] Kobidze G, Sprega G, Montanari E et al. The first LC–MS–MS stereoselective bioanalytical methods to quantitatively detect 9R- and 9S-hexahydrocannabinols and their metabolites in human blood, oral fluid and urine. *J Pharm Biomed Anal* 2024;240:115918.doi: 10.1016/j.jpba.2023.115918 38181553

[73] Bottinelli C, Baradian P, Poly A et al. Identification and quantification of both isomers of hexahydrocannabinol, (9R)-hexahydrocannabinol and (9S)-hexahydrocannabinol, in three different matrices by mass spectrometry. *Rapid Commun Mass Spectrom* 2024;38:e9711.doi: 10.1002/rcm.9711 38342829

[74] Höfert L, Becker S, Dreßler J et al. Quantification of (9R)- and (9S)-hexahydrocannabinol (HHC) via GC-MS in serum/plasma samples from drivers suspected of cannabis consumption and immunological detection of HHC and related substances in serum, urine, and saliva. *Drug Test Anal* 2024;16:489–97.doi: 10.1002/dta.3570 37652872

[75] Peters FT, Hartung M, Herbold M et al. APPENDIX B To the GTFCh Guidelines for quality assurance in forensic-toxicological analyses: requirements for the validation of analytical methods. *Toxichem Krimtech* 2009;76:185–208.

[76] Matuszewski BK, Constanzer ML, Chavez-Eng CM. Strategies for the assessment of matrix effect in quantitative bioanalytical methods based on HPLC–MS–MS. *Anal Chem* 2003;75:3019–30. doi: 10.1021/ac020361s 12964746

[77] Schwope DM, Scheidweiler KB, Huestis MA. Direct quantification of cannabinoids and cannabinoid glucuronides in whole blood by liquid chromatography-tandem mass spectrometry. *Anal Bioanal Chem* 2011;401:1273–83. doi: 10.1007/s00216-011-5197-7 21727996 PMC3159033

[78] Pichini S, Malaca S, Gottardi M et al. UHPLC–MS–MS analysis of cannabidiol metabolites in serum and urine samples. Application to an individual treated with medical cannabis. *Talanta* 2021;223:121772.doi: 10.1016/j.talanta.2020.121772 33298281

[79] Road Traffic Act Germany 2023. https://www.gesetze-im-internet.de/stvg/__24a.html (14 June 2024, date last accessed).

[80] Road Traffic Act Switzerland 2022. https://www.fedlex.admin.ch/eli/cc/2008/352/de (14 June 2024, date last accessed).

[81] Meier U, Dussy F, Scheurer E et al. Cannabinoid concentrations in blood and urine after smoking cannabidiol joints. *Forensic Sci Int* 2018;291:62–67.doi: 10.1016/j.forsciint.2018.08.009 30149280

[82] Baselt RC . Tetrahydrocannabinol. In: *Disposition of Toxic Drugs and Chemicals in Men*. 10th edn. Seal Beach, CA: Biomedical Publications, 2014, 1948.

