# Supplementary material for: Quantification of ∆9-tetrahydrocannabinol, 11-OH-THC, THC-COOH, hexahydrocannabinol, and cannabidiol in human plasma and blood by liquid chromatography–tandem mass spectrometry
Source: J Anal Toxicol. 2024 Dec 5;49(2):85–95. doi: 10.1093/jat/bkae094 (PMC11829072; doi:10.1093/jat/bkae094)
Supplement: bkae094_Supp [file bkae094_supp.zip › jat-24-4269-File004.docx]

Electronic supplementary material to the manuscript

**Quantification of ∆9-tetrahydrocannabinol (THC), 11-OH-THC, THC-COOH, hexahydrocannabinol and cannabidiol in human plasma and blood by liquid chromatography–tandem mass spectrometry**

**Table S1.** Toxicologically relevant analytes (N = 80) analyzed for interferences.

| **Analyte** | **Concentration, µg/L** | **Analyte** | **Concentration, µg/L** |
| --- | --- | --- | --- |
| 6-Acetylmorphine | 50 | 3,4-Methylenedioxymethamphetamine (MDMA) | 150 |
| 7-Aminoclonazepam | 4000 | Mephedrone | 150 |
| 7-Aminoflunitrazepam | 1000 | Methadone | 250 |
| Alprazolam | 1000 | Methamphetamine | 150 |
| Amphetamine | 150 | Methedrone | 150 |
| Benzoylecgonine | 400 | Methylecgonine | 90 |
| Benzylpiperazine | 100 | Methylone | 150 |
| Bromazepam | 4000 | Methylphenidate | 60 |
| Brotizolam | 1000 | Midazolam | 4000 |
| Buprenorphine | 250 | Mirtazapine | 150 |
| Caffeine | 250 | Morphine | 100 |
| Cannabigerol | 250 | Nicotine | 250 |
| Cannabinol | 250 | Norbuprenorphine | 250 |
| Cannabidiolic acid | 250 | Nordiazepam | 4000 |
| Citalopram | 150 | Norfentanyl | 100 |
| Clobazam | 4000 | Nortilidine | 150 |
| Clonazepam | 4000 | O-Desmethyltramadol | 400 |
| Cocaethylene | 100 | Olanzapine | 80 |
| Cocaine | 90 | Oxazepam | 10000 |
| Codeine | 200 | Oxycodone | 100 |
| Cotinine | 250 | Oxymorphone | 100 |
| Diazepam | 10000 | Pholcodine | 150 |
| Dihydrocodeine | 200 | Prothipendyl | 80 |
| 2-Ethylidene-1,5-dimethyl-3,3-diphenylpyrrolidine (EDDP) | 250 | Quetiapine | 400 |
| Ethylmorphine | 10 | Sertraline | 150 |
| Fentanyl | 10 | Tetrahydrocannabinolic acid | 250 |
| Flunitrazapam | 1000 | Tilidine | 150 |
| Hydrocodone | 100 | Tramadol | 240 |
| Hydromorphone | 100 | Trazodone | 400 |
| Lorazepam | 4000 | Triazolam | 1000 |
| 2-Methylamino-1-(3,4-methylenedioxyphenyl)butane (MBDB) | 100 | Venlafaxine | 400 |
| 3,4-Methylenedioxyamphetamine (MDA) | 100 | Zolpidem | 160 |
| 3,4-Methylenedioxyethamphetamine (MDE) | 150 | JWH-018 | 250 |
| JWH-019 | 250 | PB-22 | 250 |
| JWH-073 | 250 | 5F-PB-22 | 250 |
| JWH-081 | 250 | AKB-48 | 250 |
| JWH-122 | 250 | 5F-AB-PINACA | 250 |
| JWH-200 | 250 | AB-FUBINACA | 250 |
| JWH-250 | 250 | AB-PINACA | 250 |
| XLR-11 | 250 | HU-210 | 250 |
